# Supplementary material for: Strike one hundred to educate one: Measuring the efficacy of collective sanctions experimentally
Source: PLoS One. 2021 Apr 8;16(4):e0248599. doi: 10.1371/journal.pone.0248599 (PMC8031421; doi:10.1371/journal.pone.0248599)
Supplement: Attachment — Submitted filename: response_to_reviewers_rev2.pdf [file pone.0248599.s002.pdf]

Manuscript PONE-D-20-40936R1 “Strike one hundred to educate one: measuring the efficacy of collective sanctions experimentally”

Response to reviewers (Second revision)

Thank you for giving me the opportunity to submit a revised draft (second revision) of the manuscript .

The comments of a reviewer were very helpful. I fixed grammatical and tense problems mentioned by a Reviewer (and I have noticed and corrected a few others) as well as some inconsistencies in the bibliography. I am particularly grateful to a reviewer for referring me to a paper by Chen, Sasaki and Perc (2015) which is of fundamental importance for the collective sanctions problem, and still I somehow missed it. Now I am referring to their results, both in \`Method\` and \`Introduction\` sections.

Reviewer #1. Minor issues:

=====

> *For one, there are still some grammatical and tense problems in the revised version. For example, in page 1, line 3: “define”; in page 4, line 149-152: the format of the front quotation marks needs to be adjusted; in page 5, line 211: “N-person Prisoner’s dilemma” “N” should be italicized;*

Thank you. I fixed these forementioned and some other grammatical and syntactical errors.

=====

> *In addition, an important literature on collective punishment (implicated punishment) has not been cited. Evolution of public cooperation in a monitored society with implicated punishment and within-group enforcement. Scientific Reports, 5 (1), 1-12.*

That is an extremely valuable reference. I referred to it several times in a revised version of the paper. Thank you!

=====

> *Standing initial of the journal name in the reference should be capitalized, such as, 34-35, 40, 44-45, 49, 52.*

I checked for inconsistencies in bibliography fixing the errors mentioned by you.
